# Bidirectional Relationships and Mediating Effects Between Social Isolation, Loneliness, and Frailty in Chinese Older Adults

**DOI:** 10.1093/geroni/igae019

**Published:** 2024-02-23

**Authors:** Chaoping Pan

**Affiliations:** College of Medical Humanities and Management, Wenzhou Medical University, Wenzhou City, Zhejiang Province, China

**Keywords:** Frailty, General Cross-Lagged Panel Model, Loneliness, Social isolation

## Abstract

**Background and Objectives:**

Social isolation (SI) and loneliness are key factors that contribute to frailty among older adults. Current estimates regarding how frailty affects SI and loneliness and how SI and loneliness affect frailty may be flawed due to reverse causality. This study aimed to investigate the bidirectional relationships and mediating effects among SI, loneliness, and frailty among older adults in China.

**Research Design and Methods:**

The study analyzed data from 6 waves of the Chinese Longitudinal Healthy Longevity Survey conducted between 2002 and 2018. The sample included individuals aged 65 and older. The General Cross-Lagged Panel Model was used to account for confounding factors and reveal mediating effects.

**Results:**

Our findings specifically indicate a direct effect of SI on frailty, although suggesting that loneliness may indirectly affect frailty through its influence on SI. Additionally, frailty can lead to increased SI and loneliness.

**Discussion and Implications:**

SI and loneliness are strongly intertwined with frailty among older adults in China. To prevent the development of frailty, public health initiatives should prioritize reducing SI among older adults. Furthermore, efforts to decrease frailty levels can yield positive outcomes by mitigating both SI and loneliness among this population.

**Translational Significance:** This study investigates the bidirectional relationships and mediating effects among social isolation (SI), loneliness, and frailty among older adults in China. The results suggest that reducing SI may be an effective strategy for preventing and managing frailty among older adults in this population. Additionally, frailty can lead to increased SI and loneliness, highlighting the importance of addressing frailty in interventions aimed at reducing SI and loneliness among older adults.

Frailty is defined by a deterioration in function across various physiological systems, coupled with an increased vulnerability to stressors (1–4). Two main clinical models are commonly used to assess frailty. Fried’s phenotype model defines frailty by considering the presence of at least 3 of the following 5 criteria: slow gait speed, unintentional weight loss, self-reported exhaustion, low physical activity, and weak grip strength (5). On the other hand, Rockwood’s cumulative deficit model measures frailty using a Frailty Index (FI) that reflects the accumulation of deficits. This index is obtained by calculating the proportion of deficits present divided by the total number of deficits considered (6). It is associated with various adverse health outcomes, such as hospitalization, falls, and increased mortality risk (3–5). With the aging population increasing worldwide, frailty has emerged as a pressing public health challenge (7). Therefore, it is crucial to determine the key factors that contribute to frailty among older adults to develop interventions that can effectively address this issue and improve the health and well-being of this population.

According to the social convoy theory, social relationships can be divided into objective and subjective dimensions (8). Social isolation (SI) and loneliness are 2 dimensions of impoverished social relationships, representing objective and subjective aspects, respectively (9). SI pertains to the objective absence of social interaction with others and encompasses elements such as living alone, withdrawal from social connections, and limited engagement in social activities (10). Conversely, loneliness is a subjective experience that emerges when there is a disparity between one’s desired and actual level of social connection and relationship quality (2). Examining both SI and loneliness is important because loneliness can be entirely unrelated to SI (11). As the global population continues to age rapidly, SI has become a prominent issue among older adults. Research has revealed that between 10% and 43% of older adults experience SI in the later stages of life (12), with China reporting a notably high prevalence of 42.4% among older adults (13). Similarly, in countries like the United States and Europe, approximately 5%–40% of older adults experience feelings of loneliness (14), although China reports a loneliness rate of 31.3% among older adults (13). Both SI and loneliness have been found to negatively affect health and well-being, including outcomes such as frailty, disability, quality of life, and cognitive impairment (11,15–18).

Indeed, several studies have indicated that higher levels of SI and loneliness increase the risk of frailty among older adults (2,19,20). Although the exact mechanisms behind this association have not been extensively studied, possible explanations could include health behaviors, stress, and the maintenance and repair of physiological systems (21). Conversely, a few studies have found that health conditions may lead to increased SI and loneliness among older adults (22–24). The contribution of frailty to increased SI can be attributed to the fact that frailty often restricts the ability of older adults to engage in social activities, visit friends and family, and participate in physical activity (25). Moreover, the prediction of loneliness by frailty may be due to the fact that frailty could reduce the capacity of older adults to fulfill their socioemotional needs (26). As a result, it is possible that the relationships between SI, loneliness, and frailty are actually bidirectional in nature.

To our knowledge, there are no studies that have utilized appropriate statistical methods to explore the bidirectional relationship between SI, loneliness and frailty, and examine their potential mediating roles. Current estimates regarding how frailty affects SI and loneliness (frailty → SI, frailty → loneliness) and how SI and loneliness affect frailty (SI → frailty, loneliness → frailty) may be flawed due to reverse causality (2,19,20). Thus, methods accounting for reverse causality are necessary to establish the causal ordering among these 3 processes. It is also unclear which effect is more influential, which can affect the targeting of preventative measures. If SI, loneliness, and frailty all play important roles in bidirectional relationships, interventions targeting all 3 factors are needed to improve the health and well-being among older adults, rather than focusing on only 1 factor (20,22). Finally, failure to consider the bidirectional relationship between SI, loneliness, and frailty can result in flawed estimates of the mediating roles of these variables, which can, in turn, lead to suboptimal interventions. As a result, gaining a better understanding of the mediating role of these variables can optimize interventions targeted at intervening variables (27).

The General Cross-Lagged Panel Model (GCLM) is a powerful statistical tool that enables the examination of bidirectional relationships and mediating roles in longitudinal data. In this study, we utilized this method to analyze data from the Chinese Longitudinal Healthy Longevity Survey (CLHLS), a nationally representative survey of older adults in China conducted over a 16-year period. By utilizing this method, our aim was to investigate the directionality of the longitudinal associations between SI, loneliness, and frailty, as well as establish their mediating roles in these relationships. The hypotheses are as follows:

H1: There are bidirectional associations between SI and frailty, as well as between loneliness and frailty.

H2: It is hypothesized that SI mediates the impact of loneliness on frailty, and loneliness mediates the impact of SI on frailty. Additionally, SI mediates the impact of frailty on loneliness, although loneliness mediates the impact of frailty on SI.

## Method

### Data and Participants

This study utilized data from CLHLS, a nationally representative longitudinal survey covering 23 of the 31 provinces in China and approximately 85% of the entire population. The participants included in this data set were individuals who were 65 years of age or older. The baseline survey was conducted in 1998, and subsequent data was collected in 7 waves in 2002, 2005, 2008, 2011, 2014, and 2018. For this study, we utilized data from 6 waves of CLHLS conducted from 2000 to 2018. Further details about the CLHLS can be found elsewhere (28,29). Exclusion criteria for this study included individuals under the age of 65 at any time point, as they accounted for less than 1% of the total sample. Additionally, as this is a longitudinal study, individuals who were tracked less than twice were also excluded. Therefore, the number of respondents for each wave was: 2002, *n* = 8136; 2005, *n* = 11 427; 2008, *n* = 11 571; 2011, *n* = 9194; 2014, *n* = 6551; 2018, *n* = 3469.

Compared with the remaining participants, those who dropped out of the study exhibited certain characteristics. They were generally older, more likely to be female, had lower education levels, higher levels of loneliness, slightly lower SI scores, and higher frailty levels. More detailed information is presented in Supplementary Table 1 in the Supplemental materials. In this study, it is worth noting that there were intermittent missing values of 22.7%, 1.6%, and 11.8% for frailty, SI, and loneliness, respectively. The multiple imputation method was implemented to address potential bias due to intermittent missing data by filling in missing data. Further details on the sample characteristics and correlations of the analyzed variables can be found in Supplementary Tables 1–4.

### Measures

#### Frailty Index

FI is a well-established tool for assessing frailty and has been validated in previous research (30–32). Typically, a validated FI measures at least 30 diseases and uses an equal-weighted method to calculate scores (19,30,33,34). In our study, we utilized a comprehensive set of 45 health conditions to measure FI, which exceeded the minimum threshold and underscored the robustness of our analysis. The measurement of FI involved evaluating 8 instrumental activities of daily living, 6 activities of daily living, 6 functional limitations, 11 self-reported chronic diseases, 7 cognitive functions, 3 mental health conditions, 2 functional impairments, 1 self-rated health measure, and one investigator-rated health assessment. Each health condition was assigned a score between 0 and 1. The total FI score was obtained by summing the individual scores of all the health conditions and dividing it by 45. The final FI score ranged from 0 to 1, with higher scores suggesting greater prevalence of frailty (35).

#### Social isolation

The present study utilized the 5 dimensions recommended by previous literature (11,13,16,36,37) to measure SI in older adults. These dimensions included: (1) living alone, (2) having a spouse, (3) frequent contact with children, (4) frequent contact with siblings, and (5) participating in social activities (eg, playing cards or mahjong, joining organized social events, or working). Individuals who lived alone were without a spouse, had infrequent contact with children/siblings, or had limited social participation were coded as 1. Conversely, a value of 0 was assigned to individuals who did not live alone, had a spouse, received frequent visits from children/siblings, or engaged in social activities. The total score ranged from 0 to 5, as reported in prior research (11,16,37).

#### Loneliness

Based on previous studies (13,16,36,38,39), this study used a single item to measure loneliness, which asks “Do you feel lonely?” Responses include “never” (0 points), “hardly ever” (1 point), “sometimes” (2 points), “often” (3 points), and “always” (4 points), with a total score range of 0–4 points. The single-dimension loneliness scale is widely used in the older population, and previous research has shown that it has a high correlation with multidimensional scales (13,40).

#### Analysis

In this study, we used GCLM to analyze the data, which has been recommended by previous studies (22,41). It has been increasingly employed in social and health research, especially for investigating the associations between SI and physical function (22). One reason why GCLM is the suitable method for this analysis is its ability to model lagged relationships, which enables the exploration of bidirectional and mediating effects (22,41). This is particularly important in understanding the complex dynamics of SI, loneliness, and frailty. Additionally, GCLM offers advantages in reducing confounding effects and strengthening causal inferences by effectively controlling for both stable and time-varying factors (22,41,42).

To fit our model, we used MPlus 8 software and followed the structural equation modeling framework proposed by Zyphur et al. (41). In formal terms, the model specification used in this study is expressed as:


$$\begin{array}{l} FI_{it}=\beta_{1}SI_{it-1}+\beta_{2}FI_{it-1}+\beta_{3}Ln_{it-1}+\theta_{t}+\mu_{i}+\epsilon_{it} \end{array}$$

$$\begin{array}{l} SI_{it}=\gamma_{1}FI_{it-1}+\gamma_{2}SI_{it-1}+\gamma_{3}Ln_{it-1}+\sigma_{t}+\alpha_{i}+e_{it} \end{array}$$

$$\begin{array}{l} Ln_{it}=\mu_{1}FI_{it-1}+\mu_{2}SI_{it-1}+\mu_{3}Ln_{it-1}+\rho_{t}+\omega_{i}+\tau_{it} \end{array}$$

In the model, the subscripts i and t represent individuals and time, respectively. SI represents social isolation, FI represents Frailty Index, and Ln represents loneliness. The regression coefficients to be estimated are $\beta_{1}$, $\beta_{2}$, $\beta_{3}$, $\gamma_{1}$, $\gamma_{2}$, $\gamma_{3}$, $\mu_{1}$, $\mu_{2}$ and $\mu_{3}$. θ, σ and ρ represent time effects, although μ, α_,_ and ω capture time-invariant effects. ϵ, e_,_ and τ represent individual-specific error terms. It is important to acknowledge that the model does not incorporate specific time-varying or time-invariant variables as distinct entities. Instead, the model controls for confounding factors that can vary over time and factors that remain constant across time by treating them as collective entities. This is accomplished by including correlation terms among ϵ, e, and τ, as well as μ, α, and ω to address potential confounding. There were 2 main reasons for choosing this method to control potential confounding. First, due to limitations in the available data set, it was not possible to include all confounding variables in the analysis, which could introduce bias into the results. Furthermore, including a large number of confounding variables can lead to convergence issues with the model. Second, previous study has demonstrated that incorporating correlation terms is a more effective approach for controlling confounding factors than including specific confounding variables as covariates (41). The efficacy of this approach has been increasingly recognized and employed in other studies as well (43,44).

The cross-lagged coefficients $\beta_{1}$, $\beta_{3}$, $\gamma_{1}$, $\gamma_{3}$, $\mu_{1}$_,_ and $\mu_{2}$ are of particular interest as they reveal how differences in FI, SI, and Ln at a given time point predict differences in FI, SI, and Ln at the next time point. The autoregressive paths $\beta_{2}$, $\gamma_{2}$_,_ and $\mu_{3}$ reflect the degree to which individual differences in expected scores are predicted by differences from past time points. Furthermore, the model allows for the computation of mediating effects between SI, loneliness, and frailty by calculating the cross-lagged coefficients. Before running the analysis, the variables were standardized, resulting in the regression coefficients being expressed as standard deviation (SD) from the mean. This standardization process helps to compare different variables used in the analysis. The study employs 10 000 bootstrapping methods to compute confidence intervals. Multiple model fit indices, including the Confirmatory Fit Index (CFI), the Tucker Lewis Index (TLI), the root mean square error of approximation (RMSEA), and the standardized root mean squared residual (SRMR), were used to confirm the goodness of model fit of our analysis (41). A CFI value greater than 0.95 was deemed indicative of a good model fit. Similarly, RMSEA and SRMR values equal to or below 0.06 were considered to indicate a good fit, although values up to 0.08 were deemed acceptable (27).

## Results

At baseline, the average age of the participants was 81.83 years. Females comprised 54.8% of the sample. Around 42.5% of the participants had received at least 1 year of education, although the majority (56.1%) resided in rural areas. The participants reported a mean SI score of 2.87 and a mean loneliness score of 0.98. Frailty was assessed to have a mean score of 0.14. For more detailed information, refer to Supplementary Table 1.

Table 1 shows the goodness-of-fit statistics of GCLM on the CLHLS data set, covering waves from 2002 to 2018. The indices evaluated include CFI, TLI, RMSEA, and SRMR, with their corresponding values of 0.993, 0.988, 0.020, and 0.027, respectively. These indices indicate that the model fits the data well and provides a reliable representation of the relationships among the analyzed variables.

**Table 1.** The Goodness-of-Fit Statistics of GCLM (CLHLS, Waves 2002–2018)

Index	Values
CFI	0.993
TLI	0.988
RMSEA	0.020
SRMR	0.027

*Notes*: CFI = Confirmatory Fit Index; CLHLS = Chinese Longitudinal Healthy Longevity Survey; GCLM = General Cross-Lagged Panel Model; RMDEA = root mean square error of approximation; SRMR = standardized root mean square residual; TLI = Tucker–Lewis Index.

Table 2 displays the standardized GCLM regression coefficients and their 95% confidence intervals (CIs) for key parameters concerning frailty, SI, and loneliness over time. The analysis indicates that greater levels of SI at a given time point were linked with higher frailty in the next time point ($\beta_{1}$). Specifically, each increment of 1 SD in SI increased future frailty by 0.151 SDs (95% CI: 0.121, 0.180). Conversely, no significant association was found between loneliness at a given time point and future frailty ($\beta_{3}$).

**Table 2.** The Key Model Parameters of GCLM (CLHLS, Waves 2002–2018)

Path	Standardized coefficient (95% CI)
SI_t−1_ → SI_t_	0.378(0.354, 0.405)
SI_t−1_ → FI_t_	0.121(0.086, 0.153)
SI_t−1_ → Ln_t_	0.235(0.212, 0.254)
Ln_t−1_ → Ln_t_	0.065(0.049, 0.080)
Ln_t−1_ → SI_t_	0.051(0.038, 0.066)
Ln_t−1_ → FI_t_	0.011(−0.011, 0.032)
FI_t−1_ → FI_t_	0.349(0.292, 0.402)
FI_t−1_ → SI_t_	0.107(0.080, 0.129)
FI_t−1_ → Ln_t_	0.100(0.082, 0.117)

*Notes*: CLHLS = Chinese Longitudinal Healthy Longevity Survey; GCLM = General Cross-Lagged Panel Model.

Moreover, higher levels of frailty at a specific time point were associated with increased SI and loneliness in the next time point ($\gamma_{1}$ and $\mu_{1}$). For every increase of 1 SD in frailty, SI and loneliness increased by 0.107 SDs (95% CI: 0.080, 0.129) and 0.100 SDs (95% CI: 0.082, 0.117), respectively.

The analysis also revealed that higher levels of SI at a specific time point were associated with increased loneliness in the next time point ($\mu_{2}$ = 0.235, 95% CI: 0.212, 0.254). Conversely, higher levels of loneliness at a given time point were associated with increased SI in the next time point ($\gamma_{3}$ = 0.051, 95% CI: 0.038, 0.066). For further details, please see Table 2.

Table 3 shows the key mediating effects and their 95% CIs from GCLM analysis using data from CLHLS waves in 2002–2018. The results indicate that the path Ln_t−2_ → SI_t−1_ → FI_t_ had a standardized coefficient of 0.006 with a 95% CI of (0.003, 0.010), suggesting that SI may play a mediating role in the relationship between frailty and loneliness over time. Specifically, higher levels of loneliness at 1 point in time were associated with higher levels of SI at the following time point, which in turn was associated with higher levels of frailty in the future. However, the path SI_t−2_ → Ln_t−1_ → FI_t_ had a standardized coefficient of 0.003 with a 95% CI of (−0.002, 0.008), indicating that SI did not significantly affect frailty through loneliness.

**Table 3.** Key Mediating Effects From GCLM (CLHLS, Waves 2002–2018)

Path	Standardized coefficient (95% CI)
Ln_t−2_ → SI_t−1_ → FI_t_	0.006(0.003, 0.010)
SI_t−2_ → Ln_t−1_ → FI_t_	0.003(–0.002, 0.008)
FI_t−2_ → SI_t−1_ → Ln_t_	0.025(0.017, 0.033)
FI_t−2_ → Ln_t−1_ → SI_t_	0.005(0.003, 0.009)
SI_t−2_ → FI_t−1_ → Ln_t_	0.012(0.007,0.018)
Ln_t−2_ → FI_t−1_ → SI_t_	0.001(–0.001,0.004)

*Notes*: CLHLS = Chinese Longitudinal Healthy Longevity Survey; GCLM = General Cross-Lagged Panel Model.

Moreover, the path FI_t−2_ → SI_t−1_ → Ln_t_ had a standardized coefficient of 0.025 with a 95% CI of (0.017, 0.033), suggesting that higher levels of frailty at 1 point in time were associated with higher levels of SI in the future through loneliness of the following time point. Additionally, the path FI_t−2_ → Ln_t−1_ → SI_t_ had a standardized coefficient of 0.005 with a 95% CI of (0.003, 0.009), indicating that higher levels of frailty at 1 point in time were associated with higher levels of loneliness in the future through SI of the following time point.

Overall, these findings provide evidence for the complex interplay among frailty, SI, and loneliness over time. For more detailed information on these mediating effects, please refer to Table 3.

## Discussions

This study aimed to investigate the bidirectional associations and their mediating effects between SI, loneliness, and frailty among older adults in China using GCLM. Our findings revealed a bidirectional relationship between SI and frailty, supporting our first hypothesis. However, contrary to our initial hypothesis, we did not observe a direct effect from loneliness to frailty. Furthermore, our results indicated that SI mediates the impact of loneliness on frailty, while also mediating the impact of frailty on loneliness. Conversely, loneliness only mediates the impact of frailty on SI. These findings partially confirm our second hypothesis. These findings have important implications for understanding the complex and dynamic relationship between these 3 factors and for improving the health and well-being of older adults in China.

According to the social convoy model, SI and loneliness represent objective and subjective aspects of social relationships that may have different effects on health (8). One hypothesis explaining these different effects is that loneliness and SI may have separate pathways to influencing health (13,39). For instance, loneliness may be more likely to be associated with other risk factors, such as sleep quality (45), making it less likely to emerge as an independent risk factor. Conversely, SI may have a stronger likelihood of emerging as an independent risk factor (13). Consistent with this theory, our results indicated that higher levels of SI, but not loneliness, were independently associated with an increase in frailty among older adults. This finding is inconsistent with previous literature (19,20). Davies et al. indicated that both loneliness and SI could affect frailty among older adults (20), although Gale and colleagues only found an association between the frailty phenotype and loneliness (19). The inconsistent results may be attributed to 2 reasons. Firstly, previous studies did not adequately control for the reverse causations that exist in the relationships between SI, loneliness, and frailty, and did not adequately control for confounding factors. Secondly, differences in data sets used across studies may also contribute to the inconsistency. For instance, the mean age of our sample at baseline was 81.83, which was notably older than the samples in previous studies with mean ages of 69.3 (19) and 66.3 (20), respectively. Therefore, our study suggests that reducing frailty among older adults requires more attention to be paid to SI rather than loneliness, particularly among the oldest-old population.

Although many studies have explored the mediating effects between SI, loneliness, and mental health, few have examined their relationships with frailty (18,27). To address this gap, we conducted a mediation analysis and found that loneliness may indirectly affect frailty through SI. This suggests that loneliness has a long-term effect on frailty through SI among older adults. Our findings suggest that interventions such as community-based services, aimed at improving social connectedness, are required to reduce SI among lonely older adults and decrease their frailty levels.

Our results also revealed that health conditions can increase SI and loneliness among older adults, which is consistent with other studies (22,23). This finding further underscores the importance of addressing frailty in interventions aimed at reducing SI and loneliness among older adults. Interventions such as community healthcare services and telehealth services that aim to reduce frailty may be beneficial in reducing SI and loneliness among older adults (8,46).

Our study had several strengths. First, this study used data from a large, nationally representative sample of individuals aged 65 and older, spanning 16 years, enabling us to explore the relationships between frailty, loneliness, and SI in greater depth. Second, our study utilized a novel statistical method called GCLM, which addressed confounding by reverse causality and controlled for both observable and unobservable time-invariant and time-varying confounds. This approach strengthened causal inference and provided more accurate insights into the mediating effects. Finally, we utilized a comprehensive FI tool to measure frailty, which has been validated in previous research (30–32). This instrument takes into account various aspects of health, including physical, cognitive, and mental health, making it a more robust measure of frailty than other tools that only focus on a single dimension of health.

Our study has several limitations that should be addressed. First, the article used a single item to measure loneliness. Despite previous research showing that the single-dimension loneliness scale has a high correlation with multidimensional scales (13,40), using a composite measure could have provided a more comprehensive understanding of the relationship between loneliness and frailty. Second, due to limitations in the available data, the paper did not consider participants’ contacts with nonfamily members, such as friends or neighbors, when measuring SI. This may have limited our assessment of SI. Finally, it is important to acknowledge that attrition may introduce bias to the results, as individuals who dropped out of the study exhibited different characteristics compared with those who remained. For example, the observed null effect of loneliness on frailty could be attributed to this attrition. Future research could address the limitations identified in our study by employing more comprehensive measures of loneliness, such as multidimensional scales that capture different dimensions of loneliness. Additionally, future studies should include participants’ interactions with friends, neighbors, or other nonfamily members in the measurement of SI to validate our findings.

## Conclusion

This study investigated the bidirectional relationships and mediating effects among SI, loneliness, and frailty among older adults in China using a novel statistical method called GCLM. Our findings suggest that SI plays a more important role than loneliness in predicting frailty among older adults. Interventions targeting the reduction of SI may be necessary to improve their overall health and well-being. Moreover, we found that frailty predicts both SI and loneliness, highlighting the importance of addressing frailty in interventions aimed at reducing SI and loneliness among older adults. These findings have significant implications for improving the health and well-being among older adults and provide valuable insights into the complex relationships between these factors.

## Supplementary Material

igae019_suppl_Supplementary_Tables_S1-S4

## Data Availability

The data set used in this paper is publicly available in https://opendata.pku.edu.cn/dataset.xhtml?persistentId=doi:10.18170/DVN/WBO7LK. The study was not preregistered.
