# Supplementary material for: Bidirectional Relationships and Mediating Effects Between Social Isolation, Loneliness, and Frailty in Chinese Older Adults
Source: Innov Aging. 2024 Feb 23;8(3):igae019. doi: 10.1093/geroni/igae019 (PMC10946306; doi:10.1093/geroni/igae019)
Supplement: igae019_suppl_Supplementary_Tables_S1-S4 [file igae019_suppl_supplementary_tables_s1-s4.docx]

*Innovation in Aging* Supplemental Material: Chaoping Pan, Bidirectional Relationships and Mediating Effects between Social Isolation, Loneliness, and Frailty in Chinese Older Adults.

Table S1 Baseline characteristics of sample in 2002 for Participants who dropped out and those who remained in the study

| Variables | Mean (SD) or % | | t/χ2 | p |
| --- | --- | --- | --- | --- |
|  | Dropped out | Remained |  |  |
| Loneliness | 1.18(1.05) | 0.98(0.99) | 11.71 | <0.001 |
| Social isolation | 2.68 (0.87) | 2.87 (1.01) | -12.51 | <0.001 |
| Frailty | 0.24 (0.15) | 0.14 (0.11) | 42.94 | <0.001 |
| Age | 91.09 (10.52) | 81.83 (10.87) | -84.34 | <0.001 |
| Sex, female (%) | 61.1 | 54.8 | 44.75 | <0.001 |
| Education, received at least one year of education (%) | 34.3 | 42.5 | 112.13 | <0.001 |
| Residence, rural (%) | 52.8 | 56.1 | 29.40 | <0.001 |

Table S2 Bivariate Correlations Between frailty and SI

|  | Frailty1 | Frailty2 | Frailty3 | Frailty4 | Frailty5 | Frailty6 | SI1 | SI2 | SI3 | SI4 | SI5 | SI6 |
| --- | --- | --- | --- | --- | --- | --- | --- | --- | --- | --- | --- | --- |
| Frailty1 | 1 | 0.587 | 0.464 | 0.378 | 0.284 | 0.172 | 0.381 | 0.348 | 0.300 | 0.255 | 0.194 | 0.135 |
| Frailty2 |  | 1 | 0.570 | 0.467 | 0.382 | 0.256 | 0.337 | 0.410 | 0.345 | 0.296 | 0.233 | 0.218 |
| Frailty3 |  |  | 1 | 0.600 | 0.497 | 0.331 | 0.315 | 0.376 | 0.420 | 0.372 | 0.305 | 0.245 |
| Frailty4 |  |  |  | 1 | 0.660 | 0.425 | 0.256 | 0.306 | 0.365 | 0.396 | 0.342 | 0.269 |
| Frailty5 |  |  |  |  | 1 | 0.514 | 0.225 | 0.264 | 0.330 | 0.343 | 0.386 | 0.324 |
| Frailty6 |  |  |  |  |  | 1 | 0.152 | 0.165 | 0.232 | 0.259 | 0.287 | 0.399 |
| SI1 |  |  |  |  |  |  | 1 | 0.613 | 0.553 | 0.515 | 0.409 | 0.270 |
| SI2 |  |  |  |  |  |  |  | 1 | 0.623 | 0.575 | 0.470 | 0.328 |
| SI3 |  |  |  |  |  |  |  |  | 1 | 0.773 | 0.526 | 0.415 |
| SI4 |  |  |  |  |  |  |  |  |  | 1 | 0.600 | 0.503 |
| SI5 |  |  |  |  |  |  |  |  |  |  | 1 | 0.566 |
| SI6 |  |  |  |  |  |  |  |  |  |  |  | 1 |

Table S3 Bivariate Correlations Between frailty and loneliness

|  | Frailty1 | Frailty2 | Frailty3 | Frailty4 | Frailty5 | Frailty6 | Loneliness1 | Loneliness2 | Loneliness3 | Loneliness4 | Loneliness5 | Loneliness6 |
| --- | --- | --- | --- | --- | --- | --- | --- | --- | --- | --- | --- | --- |
| Frailty1 | 1 | 0.588 | 0.464 | 0.378 | 0.295 | 0.161 | 0.237 | 0.153 | 0.147 | 0.141 | 0.133 | 0.065 |
| Frailty2 |  | 1 | 0.571 | 0.469 | 0.382 | 0.247 | 0.118 | 0.257 | 0.163 | 0.151 | 0.143 | 0.065 |
| Frailty3 |  |  | 1 | 0.599 | 0.499 | 0.324 | 0.067 | 0.146 | 0.252 | 0.157 | 0.113 | 0.117 |
| Frailty4 |  |  |  | 1 | 0.660 | 0.413 | 0.087 | 0.092 | 0.147 | 0.264 | 0.164 | 0.142 |
| Frailty5 |  |  |  |  | 1 | 0.510 | 0.048 | 0.084 | 0.131 | 0.144 | 0.224 | 0.133 |
| Frailty6 |  |  |  |  |  | 1 | 0.044 | 0.032 | 0.082 | 0.087 | 0.049 | 0.235 |
| Loneliness1 |  |  |  |  |  |  | 1 | 0.186 | 0.137 | 0.130 | 0.131 | 0.106 |
| Loneliness2 |  |  |  |  |  |  |  | 1 | 0.179 | 0.141 | 0.157 | 0.047 |
| Loneliness3 |  |  |  |  |  |  |  |  | 1 | 0.152 | 0.117 | 0.109 |
| Loneliness4 |  |  |  |  |  |  |  |  |  | 1 | 0.232 | 0.171 |
| Loneliness5 |  |  |  |  |  |  |  |  |  |  | 1 | 0.197 |
| Loneliness6 |  |  |  |  |  |  |  |  |  |  |  | 1 |

Table S4 Bivariate Correlations Between SI and loneliness

|  | Loneliness1 | Loneliness2 | Loneliness3 | Loneliness4 | Loneliness5 | Loneliness6 | SI1 | SI2 | SI3 | SI4 | SI5 | SI6 |
| --- | --- | --- | --- | --- | --- | --- | --- | --- | --- | --- | --- | --- |
| Loneliness1 | 1 | 0.186 | 0.137 | 0.130 | 0.131 | 0.106 | 0.212 | 0.174 | 0.155 | 0.150 | 0.122 | 0.173 |
| Loneliness2 |  | 1 | 0.179 | 0.141 | 0.157 | 0.047 | 0.180 | 0.241 | 0.189 | 0.164 | 0.144 | 0.111 |
| Loneliness3 |  |  | 1 | 0.152 | 0.117 | 0.109 | 0.181 | 0.198 | 0.266 | 0.216 | 0.197 | 0.157 |
| Loneliness4 |  |  |  | 1 | 0.232 | 0.171 | 0.166 | 0.151 | 0.188 | 0.239 | 0.204 | 0.180 |
| Loneliness5 |  |  |  |  | 1 | 0.197 | 0.130 | 0.135 | 0.170 | 0.196 | 0.243 | 0.203 |
| Loneliness6 |  |  |  |  |  | 1 | 0.074 | 0.092 | 0.139 | 0.188 | 0.197 | 0.324 |
| SI1 |  |  |  |  |  |  | 1 | 0.613 | 0.553 | 0.515 | 0.411 | 0.274 |
| SI2 |  |  |  |  |  |  |  | 1 | 0.623 | 0.575 | 0.472 | 0.328 |
| SI3 |  |  |  |  |  |  |  |  | 1 | 0.774 | 0.528 | 0.417 |
| SI4 |  |  |  |  |  |  |  |  |  | 1 | 0.602 | 0.505 |
| SI5 |  |  |  |  |  |  |  |  |  |  | 1 | 0.566 |
| SI6 |  |  |  |  |  |  |  |  |  |  |  | 1 |
